# Hybrid Surgical and Catheter Treatment for Atrial Fibrillation

**DOI:** 10.1155/2013/920635

**Published:** 2013-12-16

**Authors:** Tsuyoshi Kaneko, Sary F. Aranki

**Affiliations:** Department of Cardiac Surgery, Brigham and Women's Hospital, 75 Francis Street, Boston, MA, USA

## Abstract

Advances in surgery for atrial fibrillation from cut and sew technique to thoracoscopy and new energy source have enabled minimally invasive approach which avoids median sternotomy and cardiopulmonary bypass. However, minimally invasive approach is unable to match the outcome of classic surgical technique due to difficulty in creating some of linear ablation lines. Hybrid procedure using catheter mapping and ablation in addition to minimally invasive surgical ablation has gained interest to combine the advantages of both procedures. No large study has been conducted to date comparing this new technique to other existing treatments. The aim of this paper is to review the data on hybrid procedure for atrial fibrillation and assess its early outcome and efficacy.

## 1. Introduction

Surgical treatment for atrial fibrillation (AF) has evolved over the years. Cox-Maze operation initially performed by cut and sew technique has been highly effective in the treatment of AF [1]. With recent advances in energy source to substitute classic cut and sew technique, minimally invasive technique has emerged as new approach avoiding median sternotomy and cardiopulmonary bypass [2]. Most of these procedures perform pulmonary vein isolation (PVI) and create linear lesions utilizing video-assisted thoracoscopy (VATS). However, some linear lesions cannot be created from epicardial ablation thus limiting the efficacy of this approach [3].

Catheter ablation has also evolved as effective treatment for paroxysmal AF. Linear ablation has been one of the developing fields, using 3-dimensional navigation systems for atrial mapping. This has enabled creating a similar ablation line to surgical ones. With PVI and linear ablation, success rate for single intervention is reported to be 57 to 77% [4]. However, multiple procedures are often required and have poor success rates for persistent AF and long-standing persistent AF.

Recent reports of hybrid approach which combines VATS epicardial and catheter endocardial approach, reduces each treatment's disadvantages and achieves complete ablation for high risk patients. The purpose of this paper is to review the current data on hybrid surgical and catheter ablation for AF.

## 2. Technique

Patients are considered for hybrid procedure in case of paroxysmal, persistent, or long-standing AF with left atrial dilatation over 4.5 cm based on current guidelines [5]. Preoperative transthoracic echocardiogram and computer tomography are obtained to assess PV and coronary anatomy. In addition, spirometry is required to assess pulmonary function and whether patient can tolerate selective lung ventilation. Under general anesthesia, double lumen endotracheal tube for selective lung ventilation is placed. Transesophageal echocardiogram was used to rule out left atrial thrombus.

We will describe the technique of hybrid procedure (one-step method) by Pison and associates [6] and La Meir and associates [7].

Bilateral VATS technique is the most commonly used. 12 mm camera port is placed in fifth intercostal space midaxillary line and two 5 mm ports at third and sixth or seventh intercostal space both anterior axillary line. Pericardium is opened anterior to the phrenic nerve, and blunt dissection is performed to open the transverse and oblique sinuses.

**Step 1**

Femoral vein is accessed, and transseptal puncture is performed. PV is mapped using circular mapping catheter. PVI is performed using typical energy source radiofrequency ablation (Atricure, Westchester, OH). Similar incisions are made in the left side after switching the side of single lung ventilation. The end point of pulmonary ablation is entrance and exit block. Entrance block is defined as failure to capture the PVs during pacing; exit block is defined as failure to capture when pacing from PV catheter. If sinus rhythm was obtained here, reinduction of AF is attempted by pacing coronary sinus. If AF is persistent, linear lesions are created. For severe chronic obstructive pulmonary disease, this can be performed through right VATS approach only. Left PVs are isolated using cryothermal energy balloon catheter (Arctic Front, Cryocath, Montreal, Quebec City, Canada) endocardially [8].

**Step 2**

If PVI did not eliminate AF, roof and inferior line are created epicardially connecting pulmonary veins creating a box. If entrance and exit block are not reached, conduction gaps are identified and ablated endocardially.

**Step 3**

If box did not eliminate AF, mitral isthmus ablation is performed. Ablation line starts from antrum of the left inferior PV and crosses the CS epicardially. Endocardial ablation from mitral annulus toward coronary sinus completes the ablation line. Bidirectional block is the end point in mitral isthmus ablation.

**Step 4**

If mitral isthmus ablation did not eliminate AF, inferior vena cava (IVC) to superior vena cava (SVC) and SVC circumferential lesions are added. The isolation of SVC and IVC is confirmed by testing of conduction block across the ablation lines.

**Step 5**

**Figure 1:**
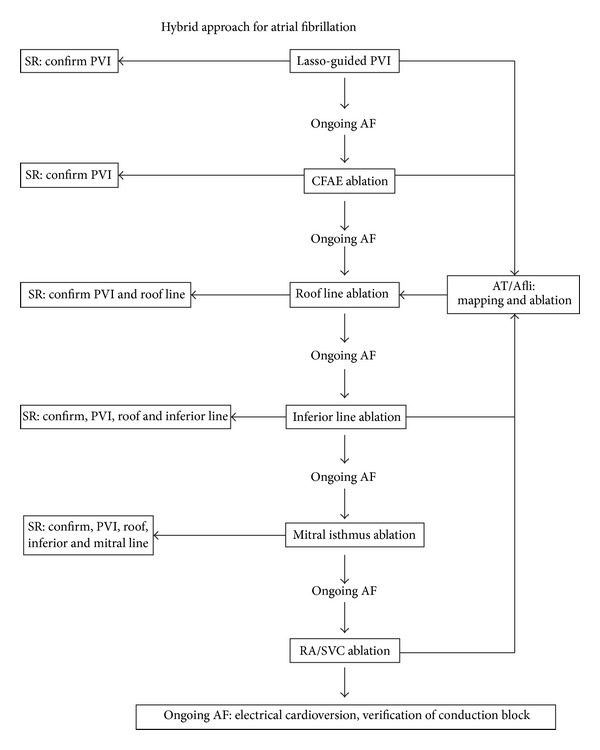
One-step approach of hybrid procedure. Reprinted from [8]. Eur J Cardiothorac Surg 2012 with permission from Oxford Journals. (AF: atrial fibrillation; SR: sinus rhythm; PVI: pulmonary vein isolation; CAFE: complex atrial fractionated electrograms; AT: atrial tachycardia; Afli: atrial flutter; RA: right atrium; and SVC: superior vena cava.)

If patient has history of typical right atrial flutter, cavotricuspid isthmus is ablated. The endpoint is bidirectional block. If patient has CHADS2 score ≥ 1, or in presence of a rapid firing coming from the left atrial appendage (LAA), and when the procedure was deemed safe, LAA exclusion/closure is performed under transesophageal echocardiographic (TEE) guidance employing a stapler (Endo GIA, Covidien, Norwalk, CT, USA) or a clip (Atricure, West Chester, OH, USA) (Figure 1).

Low molecular weight heparin is started 6 hours after procedure, and antiarrhythmic medication is restarted immediately.

## 3. Discussion

### 3.1. Surgical Treatment

Surgical treatment champed by Cox and associates has high successful rate. Cox-Maze III procedure is performed under cardiopulmonary bypass and creates complex line in the atrium using cut and sew method [1]. The success rate is reported to be over 90% [9] in experienced hands; however, due to the complexity the momentum has shifted to ablation device. New energy sources including unipolar cryoablation, unipolar microwave, unipolar laser, and unipolar radiofrequency have been used. New Cox-Maze IV procedure uses bipolar radiofrequency ablation instead of cut and sew method [10]. Bipolar energy overcomes the heat sink effect by clamping the tissue and excluding the effect of circulating blood. Cox-Maze IV is reported to have same efficacy as Cox-Maze III procedure [11]. To avoid cardiopulmonary bypass, minimally invasive technique using VATS was introduced. PVI as well as LAA removal or exclusion using radiofrequency ablation is typically performed, but PVI alone is not enough to maintain sinus rhythm. Surgical epicardial ablation has been shown to create transmural lesions around PV. The success rate is reported to range from 42 to 91% in the literature [12, 13]. The issues with surgical ablation are difficulty in creating some of linear ablation lines, the incidence of postablation flutters due to gaps in the linear lesions [14], and typical right atrial flutter from cavotricuspid isthmus which can be ablated endocardially [15].

### 3.2. Catheter Ablation

Catheter ablation is an effective method by PV, linear lesion, and autonomic ganglionated plexi ablation. However, its success rate without medication is 52%, and another procedure for completion was required in 27.3% [16]. The reason for failure is from recurrence of PV conduction, lack of transmural lesions [17], and difficulty targeting some of the ablation lines (LAA, ligament of Marshall, and epicardial ganglia) [18–20]. There have been reports of over 90% one-year success after catheter ablation, but even in these series, over 30% required repeat procedure [17]. Poor outcomes are especially seen in the presence of long-standing persistent AF [21].

### 3.3. Hybrid Procedure

Hybrid approach combines minimally invasive epicardial ablation with percutaneous endocardial ablation in step by step fashion. It enables extensive mapping to tailor the lesion set. This minimizes the disadvantages of each procedure and maximizes the advantage. Hybrid procedure was first published by Pak and associates [22]. In this report, percutaneous epicardial ablation combined with endocardial ablation was used. There have been several different types of hybrid approaches reported in the literature.

#### 3.3.1. Hybrid Procedure with Electrogram Based Mapping

Krul and associates reported their experience with 31 patients using VATS PVI and ganglionated plexus (GP) ablation guided by electrophysiological confirmation [23]. 16 had paroxysmal AF, 13 had persistent AF, and 2 had long-standing AF. 13 nonparoxysmal AF patients received additional left atrial ablation line, and 86% had no recurrence of AF at 1 year.

Han and associates had forty-five patients with AF who underwent VATS PVI, GP ablation, ligament of Marshall ablation, and LAA exclusion. After mean follow-up of 516 days, 65% had no tachyarrhythmia at 1 year [24]. They improved the success rate to 91% after performing ablation after recurrent AF or flutter.

Electrogram based mapping confirms the isolation or conduction block and adds valuable information after surgical ablation which has no feedback of the efficacy of the procedure. Fat pad in which the GPs reside is targeted using this technique. GP includes parasympathetic and sympathetic efferent neurons which play important role in triggering pulmonary vein firing [25]. Whether GP ablation needs to be done is another topic; however, electrogram based mapping enables localization of GP and ablation. GP ablation with PVI has 65% and 86% success rates at 1 year, which are not the same success rates as those of surgical maze. However, report from Han and associates found that catheter ablation after surgical ablation improved its success rate, which brings us to the next step.

#### 3.3.2. Hybrid Procedure with Postsurgical Mapping (Two-Step Approach)

Mahapatra and associates reported 15 patient experiences using surgical ablation followed by planned endocardial evaluation and catheter mapping with ablation during same hospitalization for long-standing persistent AF (two-step approach) [26]. Five patients had seven inducible flutters that were mapped and ablated. Compared to matched 30 patients who underwent catheter alone treatment following failed ablation, after mean follow-up of 20.7 ± 4.5 months, sequential treatment group had higher freedom from atrial arrhythmia both off antiarrhythmic drugs (AAD) (86.7% versus 53.3%) and on AAD (100% versus 56.7%). For the long-standing AF, success rates of surgical ablation have been 58–65% [27], and this report conveys higher success rates in a difficult patient group. The most common locations of gaps following epicardial ablation were in the roof line and line to mitral valve in this study. By performing ablation prior to discharge, they were able to avoid recurrence of AF or flutter.

Muneretto and associates reported their hybrid experience using two-step approach. Thirty-six patients with lone AF were treated [28]. Twenty-eight patients had long-standing persistent AF, and 8 had persistent AF. Intraoperative entrance and exit block were achieved in 100% and 88.8%, and 83.3% had entrance and exit block after 33 ± 2 days after the operation. Additional transcatheter lesions were performed in 61.1%. At mean follow-up of 30 months, 91.6% were in sinus rhythm and 77.7% were off AAD.

This technique allows high success rate in a difficult arrhythmia group.

#### 3.3.3. Hybrid Procedure with Simultaneous Epicardial and Endocardial Ablation (One-Step Approach)

Pison and associates reported 26 consecutive patients with AF [6]. Ten patients had persistent AF, and one had long-standing AF. They performed one-step method: simultaneous VATS epicardial approach and transvenous catheter ablation during single operation. In 23% of the patients, epicardial lesions were not transmural and endocardial touch-up was necessary. This was likely due to epicardial fat preventing transmurality. The mean length of procedure was 280 ± 84 min, and mean follow-up period was 470 ± 154 days. One-year success was 93% for paroxysmal AF and 90% for persistent AF. 9% underwent catheter ablation for recurrent AF. This result was almost equivalent to surgical Cox-Maze IV procedure of 91% one-year freedom from AF and 67% off AAD [29].

The same group compared the outcome of hybrid to that of standard VATS ablation [7]. In 35 patients with hybrid group and 28 patients with surgery-only group, hybrid group had higher sinus rhythm and off AAD at 1 year (91.4% versus 82.1%) and higher sinus rhythm and off AAD at 1 year in persistent AF (81.8% versus 44.4%). Atrial size and left atrial volume index were both reduced in both groups.

The outcomes from one-step approach are also comparable to classic surgical maze procedure although the study included more paroxysmal AF compared to two-step studies.

#### 3.3.4. Advantages of Hybrid Procedure

1. The most significant advantage of hybrid procedure is extensive mapping to tailor the lesion set.
2. It enables confirmation of unidirectional or bidirectional block to assess the efficacy of the ablation line.
3. Difficult lesion for catheter ablation (LAA, ligament of Marshall, and epicardial ganglia) can be managed epicardially.
4. Gaps during epicardial ablation can be ablated endocardially.
5. If intercaval line is added, epicardial ablation can avoid the risk of phrenic injury seen after transcatheter approach.
6. Any anatomical variation can be ruled out prior to actual surgical ablation.
7. Tamponade during transseptal puncture is avoided by direct visualization using VATS.
8. Esophageal injury from endocardial ablation can be prevented using bipolar epicardial ablation.
9. The surgical ablation device is located on the antrum of the left atrium and left as radiopaque marker and prevents PV stenosis.
10. Overall fluoroscopy time is reduced.

#### 3.3.5. Disadvantages of Hybrid Procedure

1. Overall procedure time is significantly longer.
2. Heparinization after septal puncture may increase bleeding.
3. Data on efficacy for difficult AF (persistent and long-standing) and long term freedom from AF compared to other treatment is lacking.

## 4. Conclusion

Hybrid strategy has progressed from epicardial ablation plus mapping to two-step procedure to one-step procedure. This combined surgical epicardial and catheter endocardial ablation showed high success rate compared to the outcomes of classic surgical maze procedure. Hybrid procedure will avoid sternotomy and cardiopulmonary bypass and may be the future of antiarrhythmic procedure. However, there are several obstacles to overcome. The long operative time will be constraint on surgeon, electrophysiologist, and the patient. The long term outcome is unknown, and studies showing good outcomes are of small sample size. In addition, there is lack of data for long-persistent AF which is the most anticipated patient population for this new approach. Hybrid procedure for AF is still in the early days. The expectation for this new approach is high, and larger study with longer follow-up is awaited.
